# Solid acid catalyzed carboxymethylation of bio-derived alcohols: an efficient process for the synthesis of alkyl methyl carbonates

**DOI:** 10.1038/s41598-020-69989-7

**Published:** 2020-08-04

**Authors:** Kempanna S. Kanakikodi, Sathyapal R. Churipard, A. B. Halgeri, Sanjeev P. Maradur

**Affiliations:** 1 Materials Science & Catalysis Division, Poornaprajna Institute of Scientific Research (PPISR), Bangalore, Bidalur Post-562110 India; 2 Manipal Academy of Higher Education, Manipal, 576104 India

**Keywords:** Chemistry, Materials science

## Abstract

Acid catalyzed carboxymethylation of alcohols is an emerging organic transformation that has grabbed the attention of scientific community in recent years. In the present study, sulfonated mesoporous polymer (MP-SO_3_H) is presented as a highly active solid acid catalyst to convert a wide range of alcohols into alkyl methyl carbonates. The remarkable catalytic activity of MP-SO_3_H is comparable to that of reported homogeneous acid catalysts. A good correlation was established between the catalytic activity and textural properties of the material. An exceptional catalytic activity of MP-SO_3_H was observed for DMC mediated carboxymethylation of bio-derived alcohols which is unmatchable to conventional resins and zeolites. This superior activity of MP-SO_3_H is ascribed to its intrinsic mesoporosity, high acid strength and uniform coverage of surface area by active sites. The catalyst is recyclable, resistant towards leaching and can be used in successive runs without losing the original activity. To the best of our knowledge, MP-SO_3_H is the first solid acid catalyst to exemplify highest activity for the synthesis of different alkyl methyl carbonates using DMC. The protocol developed herein opens up new avenues to transform wide range of bio-alcohols into useful organic carbonates in the future.

## Introduction

The global demand for energy production is increasing at an alarming rate and it is imperative to switch over to the bio-based energy resources. Biomass is the promising and renewable alternative for the conventional energy resources to develop sustainable processes^1^. Among many biomass valorization routes, bio-ethanol is the potential candidate and can be used as the renewable building block for the production of higher alcohols by means of guerbet process^2^. These alcohols undergo carbonate interchange/carboxymethylation reaction with dimethyl carbonate (DMC) to produce alkyl carbonates/carbonate esters. The DMC is the most studied reagent for the carboxymethylation of various alcohols ascribed to its green credentials such as sustainable synthesis, non-toxic, biodegradable and noncorrosive^3–5^. The green features of dimethyl carbonate (DMC) make it more valuable chemical from both academic and industrial standpoints^6–10^. Lately, many materials have been reported for the green synthesis of DMC using greenhouse gas like CO_2_ with methanol^11–21^. Owing to its beneficial properties, DMC has got a number of applications as green solvent in pharmaceutical synthesis and bio-catalysis. On top of it, DMC is a safe substitute for the highly toxic traditional chemicals such as dimethyl sulfide, methyl halides and phosgene in the alkylation and carboxymethylation reactions^22–26^.

**Scheme 1:**
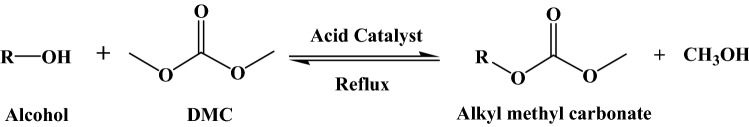
Reaction scheme for DMC mediated carboxymethylation.

DMC undergoes carboxymethylation/carbonate interchange reaction (CIR) with various alcohols to yield alkyl methyl carbonates or dialkyl carbonates without generating inorganic salts as the byproduct (Scheme 1). These organic carbonates have got numerous applications such as nonprotic solvents, lubricating oils, fuel additives and key intermediates to synthesize polycarbonates, plasticizers, monomers for organic glasses, atomoxetine, roccellaric acid, herbicides, fungicides, pesticides and nephrosteranic acid^23,24,27–32^. The reduced environmental footprints and replacement of various hazardous chemicals by organic carbonates make them one of the strong contenders to meet guidelines of green chemistry. In addition, the organic carbonates are spotlighted as the possible alternatives for VOCs (volatile organic compounds)^4,33^. Currently, most of the available reports for the carboxymethylation of various alcohols with DMC are base catalyzed. The applicability of base catalysts is limited and cannot be employed for the acidic substrates due to Lewis acidic nature of the substrates that leads to the lower catalytic activity^22^.

Numerous catalytic materials are reported for the DMC mediated carboxymethylation of various alcohols. Stanley et al. reported K_2_CO_3_, CsF/α-Al_2_O_3_, trioctylmethylphosphonium methyl carbonate ([P_8881_][CH_3_OCOO]), NaX Zeolite, and NaY zeolite: Wherein, K_2_CO_3_ and ([P_8881_][CH_3_OCOO]) showed good activity to upgrade alcohols of lignin building block^34^. Abbas-Alli et al. investigated the tin and titanium based materials such as *n*-Bu_2_SnO, *n*-[Bu_2_Sn(OPh)]_2_, Ti(OBu)_4_, etc. The conversions were lower but the authors were successful for selective synthesis of diphenyl and diaryl carbonates^35^. Tabanelli et al. evaluated the catalytic activity of NaOMe, trioctylmethylphosphoniummethylcarbonate (P_8881_-MC) and MgO catalysts for the synthesis of aliphatic alkyl methyl carbonates and catechol carbonate^36^. Khusnutdinov et al. transformed various aliphatic and aromatic alcohols into alkyl methyl ethers and alkyl methyl carbonates over Co_2_(CO)_8_ and W(CO)_6_ catalysts^23^. Fiorani et al. developed the straightforward protocol for the efficient synthesis of dibenzyl carbonate over CsF/α-Al_2_O_3_ and ([P_8881_][H_3_COCO_2_]) catalysts. The CsF/α-Al_2_O_3_ demonstrated the good catalytic activity with 70% yield of dibenzyl carbonate^37^. Ramesh et al. investigated the catalytic activity of spray dried-NaAlO_2_ catalyst for the carboxymethylation of different aromatic alcohols^24^. Wherein, the material surpassed the catalytic activity of various conventional materials like Na-X, Na-Y, MgO, hydrotalcite and NaOH etc. for the synthesis of cinnamyl methyl carbonate. Carloni and co-workers reported the MCM-41-TBD hybrid organic–inorganic material for the synthesis of carbamates and unsymmetrical carbonates^14,38^. Kenar et al. investigated the catalytic activity of dibutyl tin oxide (Bu_2_SnO) catalyst for carbonate interchange reaction of various linear and branched alcohols with diethyl carbonate. Herein, the reactions were carried out in the range of 110 °C to 140 °C to achieve 70% to 80% yield of dialkyl carbonate esters with minimal amount of catalyst^11,25^. Hatano et al. developed acid–base combined homogeneous catalysts for the efficient transesterification of 1-ethynyl-1-cyclohexanol with DMC. Here, lanthanum(III) nitrate alkoxide catalyst with different additives such as [PMe(octyl)_3_](OCO_2_Me) and P(octyl)_3_ has performed well to achieve > 98% yield of the alkyl methyl carbonate^39^. Cattelan et al. explored the catalytic activity of Mg–Al hydrotalcites for the valorization of OH-bearing bio-derived chemicals with non toxic DMC and DEC (Diethyl carbonate). The biomolecules such as glycerol formal, solketal, glycerol carbonate, furfuryl alcohol and tetrahydrofurfuryl alcohol have been successfully transformed with good yield of desired carbonate in continuous flow system^40^. In addition, the other base catalysts such as CsCO_3_^41^, nanocrystalline-MgO, MgLa mixed oxides^15,29^, K_2_CO_3_^42,43^, KW2000, basic Al_2_O_3_, acidic Al_2_O_3_^28,38^, KF/α-Al_2_O_3,_ KF/TiO_2_, CsF^27^, 1,8-diazobicyclo[5.4.0]undec-7-ene (DBU)^44^ etc have also been reported for DMC mediated carboxymethylation of various alcohols.

The thorough literature survey suggests that the most DMC mediated carboxymethylation of alcohols are base catalyzed and very limited literature on acid catalysts. The series of homogeneous base catalysts reported are associated with the conventional problems of homogeneous catalysis. The heterogeneous base catalysts are in forefront to circumvent the limitations of the homogenous catalysts for carboxymethylation of alcohols^45^. The heterogeneous base catalysts demonstrated the good catalytic activities for carboxymethylation of alcohols with DMC but at the expense of harsh reaction conditions. In addition, the base catalysts cannot be used to transform acidic substrates into corresponding carbonates. The acid mediated carboxymethylation is highly desirable to extend the applicability of the catalysts to acidic substrates. Bernini et al. investigated the activity of H_2_SO_4_ to transform alcoholic chain of phenols into carbonates^44^. Saimeng Jin et al. studied the catalytic activity of Brønsted and Lewis acidic catalysts for DMC mediated carboxymethylation^6,22^. Chevella et al. investigated the catalytic activity of nano-crystalline ZSM-5 zeolite for the carboxymethylation of various alcohols with DMC^46^. To the best of our knowledge, these are the only four reports on acid catalyzed carboxymethylation of alcohols. Despite of the significant research efforts, to our surprise only one heterogeneous acid catalyst (Nano-ZSM-5) was reported recently for this reaction. However, the reported yields of carbonates are achieved at the expense of harsh reaction conditions such as high catalyst concentration (> 110wt%), mole ratio (Alcohol:DMC =  > 23), reaction temperature (110 °C), reaction time (24 h and more in some cases) and sealed tube operation. On the other hand, we have developed simple and efficient approach for the carboxymethylation of various alcohols with DMC over MP-SO_3_H which is greener in contrast to the first solid acid reported for this reaction. The mesoporous polymers are the new class of materials having organic backbone and mesoporous architecture. The polymers can be used as the support for ionic liquids or as the catalysts in many organic transformations^47–50^.In this study, sulfonated mesoporous polymer (MP-SO_3_H) is presented as the best fitting candidate to transform various alcohols into corresponding alkyl methyl carbonate esters. The systematic study of different reaction parameters suggests that MP-SO_3_H is a potential catalyst to demonstrate more than 93% conversion of alcohols with > 95% selectivity to alkyl methyl carbonates together with high recycling efficiency. To the best of our knowledge, MP-SO_3_H is a first solid acid catalyst to demonstrate high yield of alkyl methyl carbonates in DMC mediated carboxymethylation of various alcohols under mild reaction conditions.

## Results and discussion

The surface morphology and sulfur mapping of sulfonated mesoporous polymer (MP-SO_3_H) has been recorded on SU3500 SEM–EDX instrument. The SEM and SEM–EDX micrographs of MP and MP-SO_3_H are shown in Figure S1. The scanning electron microscopy analysis of MP-SO_3_H reveals that the particles have irregular morphology with disordered shapes and the particle sizes are in the range of 2 µm to 40 µm. The elemental mapping of sulfur displays the uniform distribution of ‘S’ atoms on the polymeric network. The uniform coverage of the surface area by active sites plays a vital role in achieving good catalytic activity.

The specific surface area, pore size distribution, pore volumes of all the polymeric materials were determined using BELSORP-mini-II instrument at liquid nitrogen temperature (− 196 °C). Prior to the N_2_ physisorption measurement, the polymeric materials were out gassed under vacuum for 2 h at 393 K. All the polymeric materials exhibits type IV isotherms with hysteresis loop which is the symptomatic feature of mesoporous materials. Herein, the pronounced hysteresis loop is observed due to the capillary condensation of nitrogen in the mesopores of the polymeric materials (Figure S2)^51,52^. The unfunctionalized polymer (MP) demonstrated very high surface area, pore size and pore volumes. However, the accretion of the –SO_3_H groups lead to decrease in the specific surface area, pore volume and pore sizes in the MP-SO_3_H polymer. The reduction in the porosity of mesoporous polymers is attributed to the anchoring of high molecular weight –SO_3_H sites over the network of MP. In addition, the accumulation of –SO_3_H groups on to the polymeric network imparts hydrophilicity in the material which is evident from the contact angle measurement images are shown in Figure S3. BJH plot demonstrated the wide pore size distribution in the mesoporous range and all physico-chemical properties are depicted in the Table 1.

**Table 1:** Physico-chemical properties of MP and MP-SO_3_H.

Sample	S_BET_ (m^2^/g)	V_t_^a^ (ccg^−1^)	d_av_ (nm)	S content^b^ (mmol/g)	Acidity (mmol/g)^c^
MP	653	1.28	20.2	–	–
MP-SO_3_H-4	422	0.79	8.1	1.55	1.7
MP-SO_3_H-8	417	0.75	7.9	1.73	1.9
MP-SO_3_H-12	409	0.73	7.6	1.83	2.0
MP-SO_3_H-24	399	0.71	7.6	2.10	2.3

^a^From BJH method, ^b^taken from reference^53^, ^c^measured by acid–base titration, d_av_ = average pore diameter from BET.

The FTIR spectra of MP-SO_3_H were recorded on alpha-T Bruker spectrophotometer having Si–C source. The FTIR analysis has been carried out in a transmittance mode in the range 450 cm^−1^ to 4000 cm^−1^ (Fig. 1.1). FTIR analysis enables us to confirm the successful anchoring of –SO_3_H groups on to the porous network of the polymer. The absorption bands marked at 1016 cm^−1^ and 1173 cm^−1^ are assigned to the symmetric and asymmetric stretching vibrations of O=S=O bonds present in the –SO_3_H group anchored on the MP-network. The peak observed at 1043 cm^−1^ has been assigned to C–S stretching frequency which confirms the presence of –SO_3_H groups in the polymer network. The peaks assigned at 2959 cm^−1^ and 2927 cm^−1^ indicates the saturated and unsaturated C–H stretching vibrations of polymeric backbone respectively. The characteristic bands at 625 cm^−1^ and 3433 cm^−1^ are assigned to bending and stretching frequencies of O–H group present in the –SO_3_H. The characteristic stretching and bending vibrations mentioned above indicates the successful anchoring of –SO_3_H on to MP-network. The FTIR analysis of all MP and MP-SO_3_H materials are depicted in Figure S4^49,54–57^.

The thermal stability of the polymeric materials has been evaluated by thermogravimetric analysis (TGA). The polymeric material (MP-SO_3_H-8) showed three weight losses at different regions of the TGA curve (Fig. 1.2). A weight loss of about 15% was observed below 110 °C for MP-SO_3_H-8 catalyst as a result of desorption of water molecules adsorbed on the surface of polymers. The accretion of –SO_3_H groups on the polymer network imparts hydrophilicity in the polymer. The successive weight loss from 210 to 380 °C is associated with the decomposition of –SO_3_H groups present in the polymeric network. Herein, this weight loss indicates that the active sites are stable compared to the conventional resins and can be employed in a reaction involving harsh conditions. The final weight loss in the region 400 °C to 580 °C is ascribed to the rupture of polymeric bonds. The TGA analysis of all the polymeric materials has been evaluated by thermogravimetric analysis and depicted in Figure S5.

The transmission electron microscopy (TEM) analysis was performed for MP-SO_3_H-8 in order to confirm the porosity of the material. The TEM micrographs were recorded at different magnifications to visualize the mesoporosity in the MP-SO_3_H. The recorded TEM micrographs confirm the presence of mesoporosity in the range of 3 nm to 16 nm. The pore dimensions obtained from TEM analysis are in good agreement with the data obtained from the N_2_ sorption studies. TEM-EDS analysis has been performed at 1 µm magnification to understand the mode of distribution of active sites over the polymer network. The EDS analysis of ‘S’ atom showed the homogeneous coverage of the surface area by active sites (Fig. 1.3). The TEM-EDS and SEM–EDS elemental mapping studies proven that the active sites are uniformly distributed over the high surface area of the material which plays a vital role in the alkyl methyl carbonates synthesis. The transmission electron microscopy analysis of all the materials are shown in Figure S6. The TEM analysis results obtained for all the materials are in accordance with the data obtained from BET surface area measurement. The X-ray analysis has been performed for MP-SO_3_H-8 and it does not show any characteristic peaks but a broad peak around 20° indicates that the material is amorphous (Figure S7).

The ^13^C NMR spectrum of MP-SO_3_H-8 was recorded to confirm the structure and functionality of the material (Fig. 1.4). The strong signal marked at 41 ppm is due to the aliphatic carbon chain of polydivinylbenzene network. Signal at 143 ppm is associated with the C–S bond present in the polymer. This chemical shift clearly indicates the successful sulfonation of aromatic ring. The small signal marked at 138 ppm is due to the residual vinyl groups present in the material which in turn indicates the high cross link density of the material. On top of it, the chemical shift at 41 ppm is very strong indicating high cross link density of material^56^.

The MP-SO_3_H has been characterized by XPS analysis to know the surface elemental composition of the material (Fig. 1.5). The XPS spectra of the material exhibits the characteristic signals of C, O, and S elements present in the MP-SO_3_H. The signals associated with the S element are observed at 169.5 eV and 233.5 eV which are of S2p and S2s electrons respectively (Fig. 1.5a,b). These signals are assigned to S–O and S =O bonds present in sulfonic group of the polymer catalyst. The binding energy peaks around 284.84 eV and 286.4 eV are associated with the C–C and C–S bonds of the material respectively (Fig. 1.5c). These characteristic binding energies confirms the functionalized sulfonic groups. The XPS analysis and FTIR data are in good agreement with each other and confirms the functionalization of polymer with sulfonic groups. The surface composition of the polymer catalyst MP-SO_3_H-8 Has been given in the Table S1.

**Figure 1:**
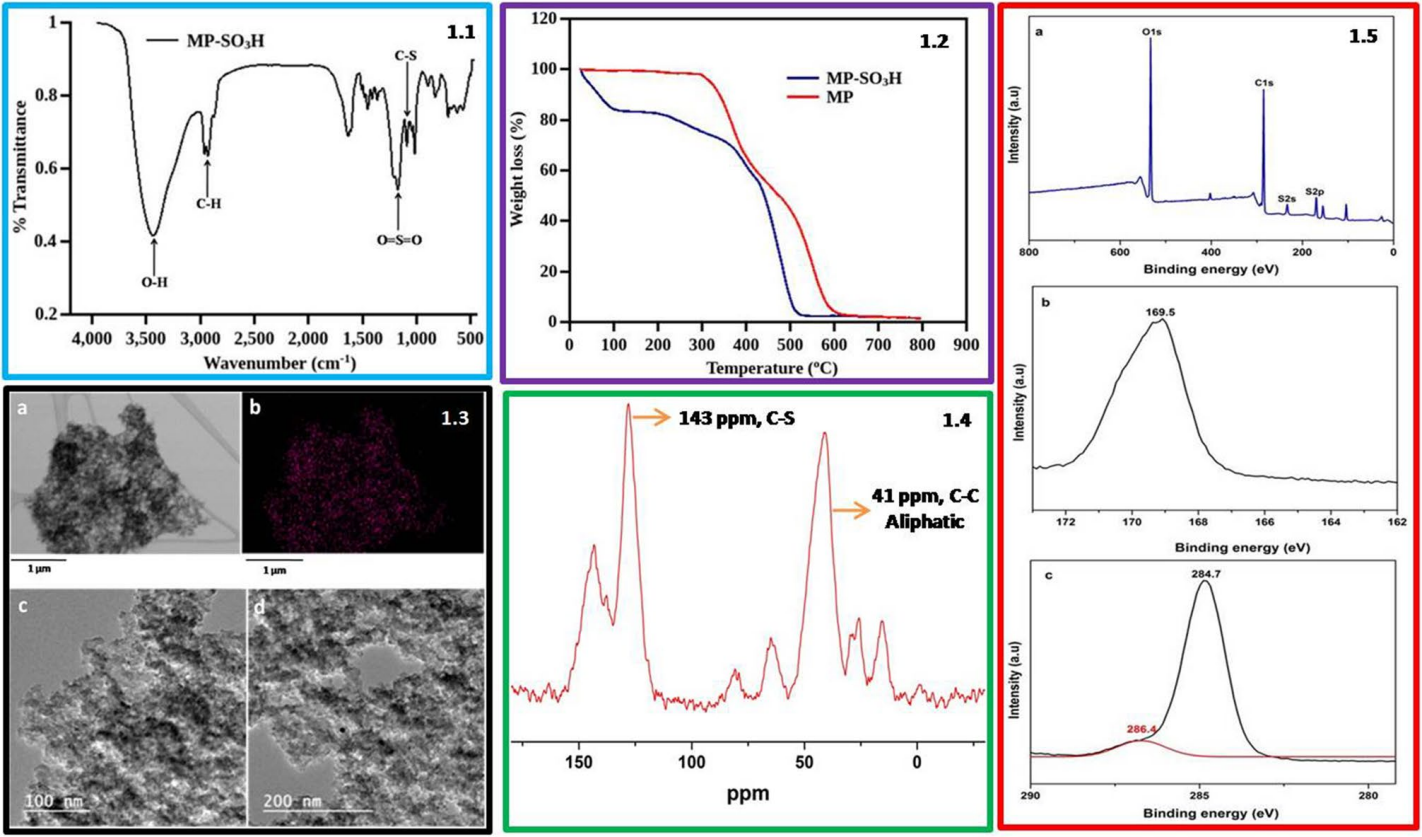
Characterization data of MP-SO_3_H, (**1.1**) FTIR spectra of sulfonated mesoporous polymer (MP-SO_3_H-8), (**1.2**) Thermogravimetric analysis plot of MP and MP-SO_3_H-8, (**1.3**) (a) and (b) TEM-EDS images of MP-SO_3_H-8, (c) and (d) transmission electron micrographs of MP-SO_3_H-8, (**1.4**): ^13^C MAS NMR spectrum of MP-SO_3_H-8, (**1.5**): XPS measurements (a) XPS survey, (b) binding energy peak of S2p, (c) binding energy peak of C–C and C–S.

The acidity present in MP-SO_3_H-X and Amberlyst-15 is measured by acid–base titration. The acidity values of sulfated zirconia, and HBEA are taken from the literature (Table S2)^53^. The CHNS elemental analysis of the polymeric catalysts has been performed to quantify sulfur content in the catalyst and compared it with the acidity values obtained from titrations. The amount of sulfur present in the polymer catalyst is varied from 1.5 to 2.1 mmol/g by varying the sulfonation time from 4 to 24 h. The acidity of the materials obtained from CHNS elemental analysis and acid–base titrations are in good agreement with each other (Table 1). In order to determine the strength of acidic sites in the MP-SO_3_H catalysts potentiometric titrations were performed by exchanging all the catalysts with 0.1 N NaNO_3_ solution. The exchanged solution was potentiometrically titrated against standardized 0.02 N NaOH solution. The pKa values were found to be 1.94, 1.93, 1.89 and 1.90 for MP-SO_3_H-4, MP-SO_3_H-8, MP-SO_3_H-12 and MP-SO_3_H-24 respectively. The pKa values obtained by the potentiometric titrations suggest that all the MP-SO_3_H catalysts have similar acidic strength.

## Catalytic activity

The synthesized catalysts were screened for DMC mediated carboxymethylation of butanol such as MP-SO_3_H, Amberlyst-15, SO_4_^–2^/ZrO_2_, HBEA and H_2_SO_4_ to produce butyl methyl carbonate (Table 2). Among the screened acid catalysts, the MP-SO_3_H catalysts demonstrated the highest yield of alkyl methyl carbonate. With increase in acidity of the material from 1.7 mmol/g (MP-SO_3_H-4) to 1.9 mmol/g (MP-SO_3_H-8) we could observe slight increase in the alcohol conversion. However, the further increase in the acidity of the material showed the decrease in conversion of butanol. This is attributed to decrease in the surface area of MP-SO_3_H polymer at high –SO_3_H content which results in decrease of accessible active sites leading to lower catalytic activity. The catalytic activity of MP-SO_3_H-8 is compared with the conventional heterogeneous catalysts mentioned herein. Amberlyst-15 demonstrated the good alcohol conversion (58%) and selectivity towards the butyl methyl carbonate. However, the catalytic activity of Amberlyst-15 is still lower compared to the MP-SO_3_H-8 (92% alcohol conversion). In addition, HBEA and sulfated zirconia suffers from the lower conversion of the alcohols. The lower activity of these materials could be attributed to the lower surface area, lower acidity and micro porosity of these materials (Table S3). The homogeneous reaction was performed under similar reaction conditions using H_2_SO_4_ (5 mol%) and compared with the MP-SO_3_H-8. The reactions were repeated to check the standard deviation in the results and are tabulated in the Table S4. The activity of MP-SO_3_H-8 is comparable with the homogeneous acid catalysts reported and can be used for efficient conversion of various alcohols. These observations suggest that the material with uniform coverage of surface area by active sites, intrinsic mesoporosity and optimum acidity with good strength will be of choice to achieve high selectivity and conversions. The MP-SO_3_H-8 having optimum sulfonic groups surpassed the catalytic activity of all the materials screened and the reaction parameters were further optimized for the same catalyst.

**Table 2:** Catalyst screening.

Catalyst	Butanol conversion (mol%)	BMC selectivity(mol%)
MP-SO_3_H-4	87.6	96.0
MP-SO_3_H-8	91.8	95.9
MP-SO_3_H-12	86.1	96.1
MP-SO_3_H-24	84.0	96.0
Amberlyst-15	57.7	96.2
SO_4_^–2^/ZrO_2_ (2 N)	4.3	100.0
HBEA (25)	22.1	100.0
H_2_SO_4_	89.7	97.1

Reaction conditions: catalyst concentration—5 mol% (w.r.t Butanol), mole ratio—1:10 (butanol:DMC), reflux condition, reaction time—24 h, conversions were calculated with respect to the limiting reagent (Butanol), BMC (butyl methyl carbonate).

**Figure 2:**
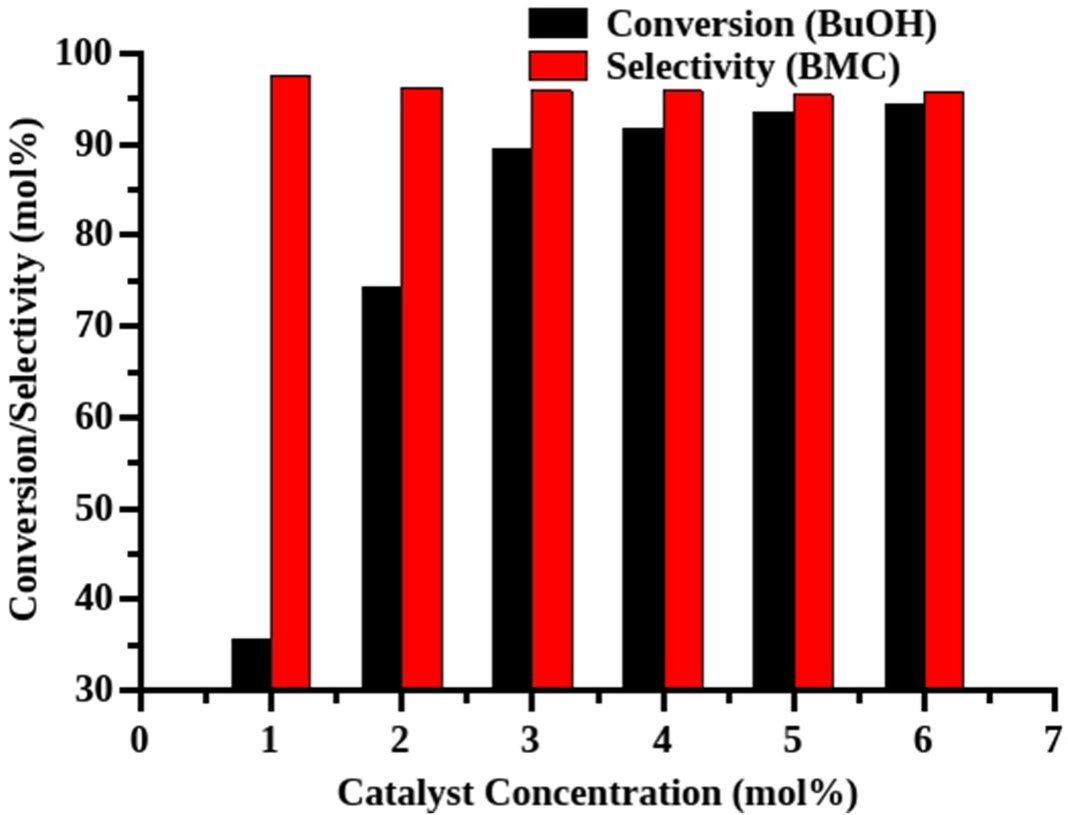
Effect of catalyst loading. Reaction conditions: Catalyst—MP-SO_3_H-8, butanol:DMC—6:60 mmol, reaction time—24 h, conversions were calculated with respect to the limiting reagent (butanol), reflux condition, BMC (butyl methyl carbonate).

The effect of catalyst concentration on the alcohol conversion and selectivity was studied systematically. The catalyst concentration was varied from 1 to 7 mol% with respect to limiting reagent (butanol). It is observed that the percentage conversion of butanol increased from 35 to 92% with increase in catalyst concentration from 1 to 5 mol%. The observed increase in the alcohol conversion is attributed to the addition of extra active sites which are responsible for converting more alcohol molecules. Further increase in the catalyst concentration to 6 mol% and 7 mol% showed only a marginal increase in the alcohol conversion (Fig. 2). Therefore the optimum catalyst loading of 5 mol% is essential to achieve more conversion and good selectivity to butyl methyl carbonate (BMC).

**Figure 3:**
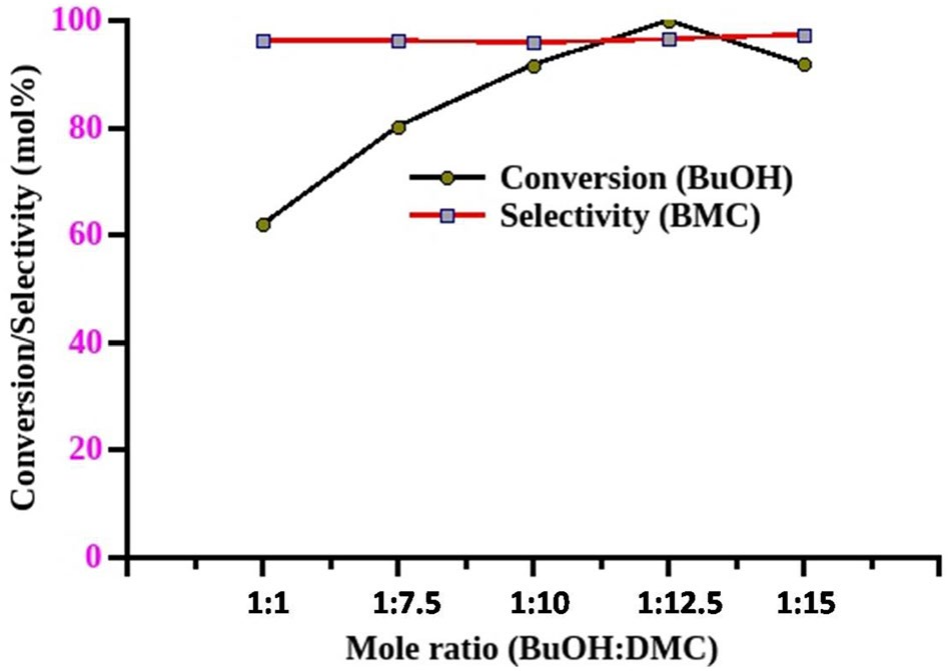
Effect of mole ratio between the reactants. Reaction conditions: Catalyst—MP-SO_3_H-8, catalyst concentration—5 mol% (w.r.t butanol), reaction time—24 h, conversions were calculated with respect to the limiting reagent (butanol), reflux condition, BMC (butyl methyl carbonate).

The stoichiometry between the reactants in any chemical transformation plays a vital role to get desired conversion and selectivities. The effect of mole ratio between butanol and DMC has been investigated for DMC mediated carboxymethylation of butanol using 5 mol% of MP-SO_3_H-8 catalyst by keeping other reaction parameters constant. The mole ratio between butanol and DMC was varied from 1:5 to 1:15. The conversion of butanol increased from 62 to > 99% by increase in mole ratio from 1:5 to 1:12.5 but the selectivity remains same in all the cases (Fig. 3). Further increase in mole ratio to 1:15 leads to decrease in conversion which is ascribed to the dilution of butanol by DMC. Thereby the adsorption of butanol will be prevented and leads to less conversion of alcohol^48^. The effect of mole ratio study confirms that the ratio 1:12.5 between butanol and DMC is found to be the best and used for the optimization of other reaction parameters.

**Figure 4:**
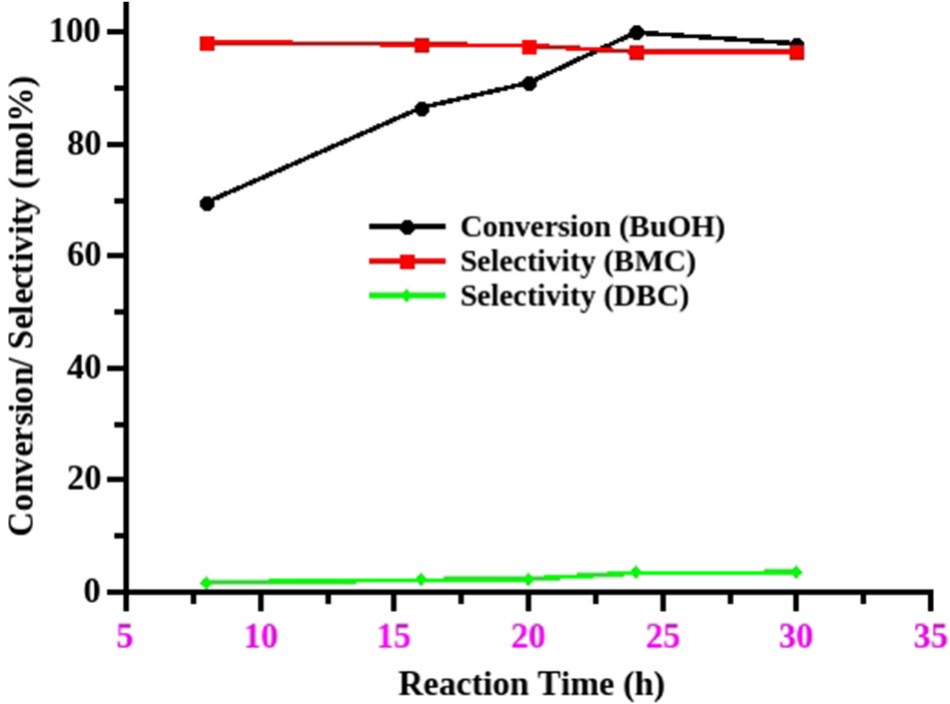
Effect of reaction time. Reaction conditions: Catalyst—MP-SO_3_H-8, catalyst concentration—5 mol% (w.r.t Butanol), mole ratio—1:12.5 (butanol:DMC), conversions were calculated with respect to the limiting reagent (butanol), reflux condition, BMC (butyl methyl carbonate), DBC (dibutyl carbonate).

The effect of reaction time was studied for the DMC mediated carboxymethylation of butanol to identify the optimum reaction time to achieve the maximum conversion and selectivity. The amount of catalyst (MP-SO_3_H-8, 5 mol%), mole ratio (1:12.5) and reaction temperature (90 °C) are kept unchanged to study the effect of time. The five independent reactions were performed to examine the effect of reaction time for carboxymethylation of butanol with DMC. The increase in reaction time from 8 to 24 h exemplified the increase in butanol conversion from 70 to > 99%. These results show that the material is really efficient to convert more than 70% of butanol within 8 h. The prolongation of reaction time to 30 h is not beneficial to achieve more selectivity. The study showed that 24 h is the optimum time to achieve more selectivity at maximum conversion of alcohol (Fig. 4).

**Figure 5:**
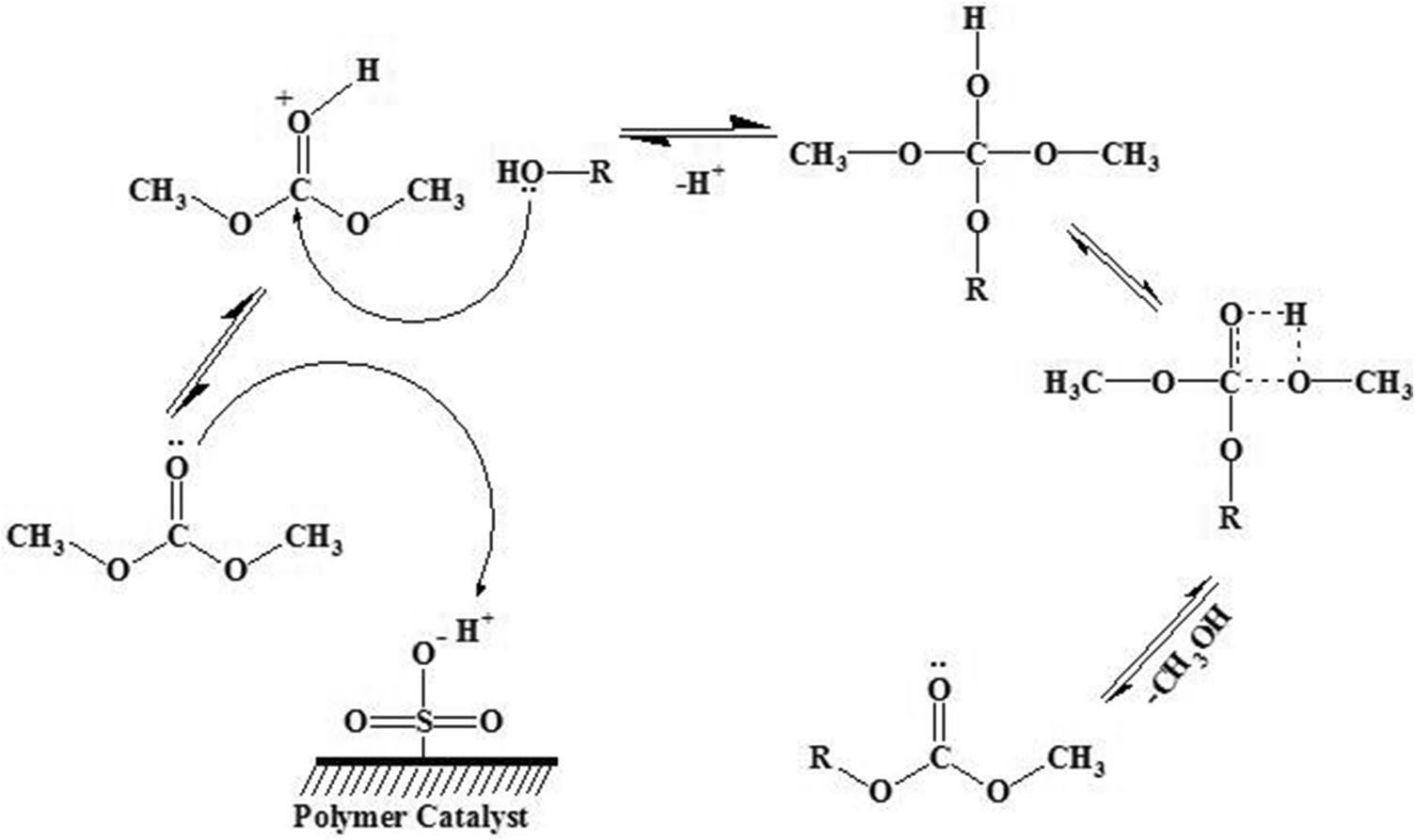
Plausible reaction mechanism for acid catalyzed carboxymethylation.

The acid catalyzed carboxymethylation of alcohols with DMC proceeds via transesterification mechanism^6,22^. The lone pair of electrons present on the carbonyl group of DMC picks up the proton from the solid acid. Then the carbon atom of DMC undergoes nucleophilic attack from the alcohol. The hemiketal intermediate is formed by the elimination of proton. This hemiketal intermediate eliminates methanol and leads to the formation of alkyl methyl carbonate product (Fig. 5). The reaction mixture is analyzed by the gas chromatograph and confirmed by GC-mass analysis (Figure S8).

**Figure 6:**
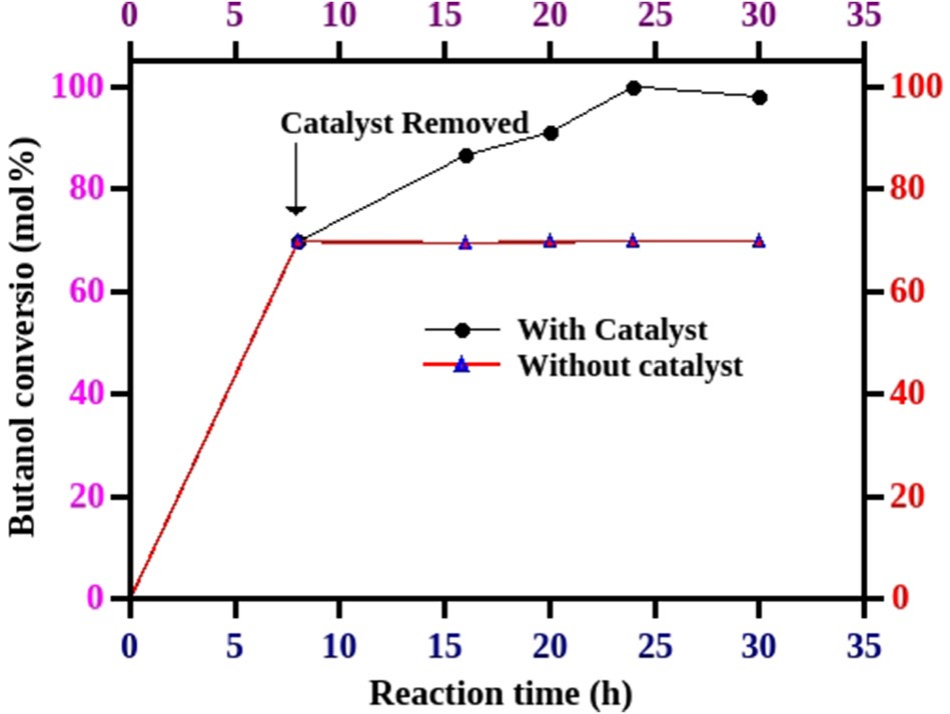
Leaching studies. Reaction conditions: Catalyst—MP-SO_3_H-8, catalyst concentration—5 mol% (w.r.t Butanol), mole ratio—1:12.5 (butanol:DMC), reaction time—8 h, conversions were calculated with respect to the limiting reagent (butanol), reflux condition.

**Figure 7:**
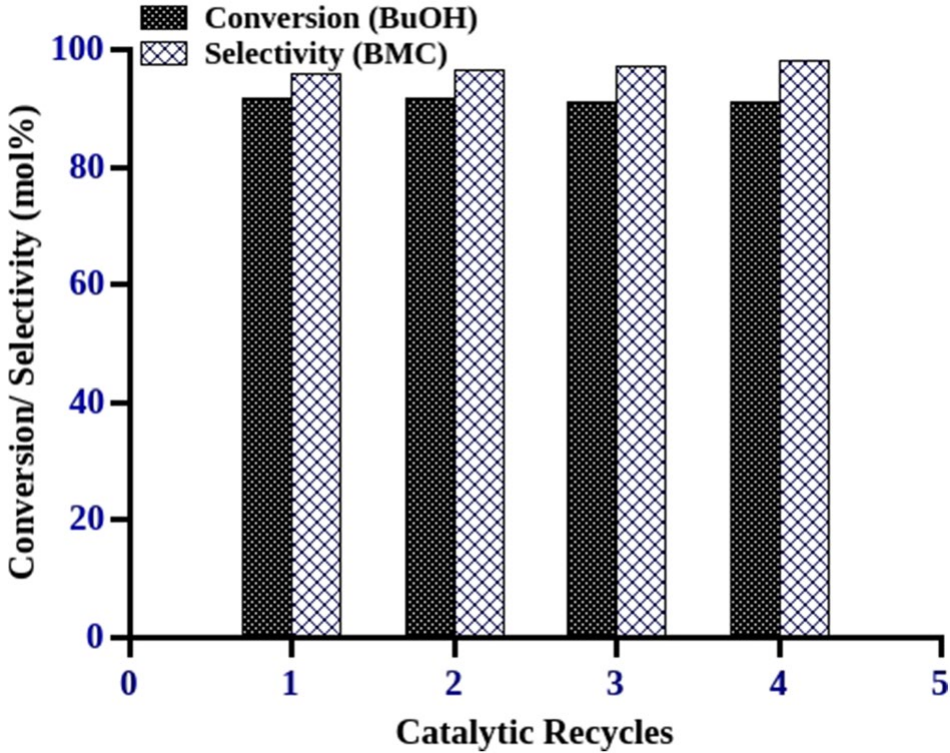
Recyclability studies. Reaction conditions: Catalyst—MP-SO_3_H-8, catalyst concentration—5 mol% (w.r.t Butanol), mole ratio—1:10 (butanol:DMC), reaction time—24 h, conversions were calculated with respect to the limiting reagent (butanol), reflux condition, BMC (butyl methyl carbonate).

The hot filtration experiment was performed to check the stability of the active sites towards leaching in the reaction medium. After 8 h of reaction time, the catalyst was separated by hot filtration and filtrate was allowed to react at the similar reaction temperature. The samples were withdrawn at different reaction times (8 h, 16 h, 20 h and 24 h) and quantified by gas chromatograph. There is no increase in butanol conversion after catalyst removal which indicates that there is no leaching of active sites during the course of reaction (Fig. 6). The catalyst recycle experiments were performed under identical reaction conditions. After each run, the catalyst was washed with excess of methanol and dried in hot air oven for 2 h at 100 °C. The dried catalytic material was employed for the next catalytic run under similar reaction conditions. The catalyst reusability experiments were performed up to three recycles and MP-SO_3_H-8 demonstrated the similar catalytic activity as the fresh catalyst in all the experiments (Fig. 7). The study shows that the material is resistant towards leaching and recyclable which can be used repeatedly without losing its catalytic activity.

The general applicability of MP-SO_3_H-8 was studied for various alcohols involving primary, secondary, tertiary and aromatic alcohols. The MP-SO_3_H-8 affords > 95% conversion of primary alcohols to corresponding alkyl methyl carbonates with very high selectivity at 24 h reaction time. The conversion of secondary alcohols is moderate (35–45%) at 24 h and no conversion of the tertiary alcohols was observed. The phenomenon could be attributed to the formation of stable intermediate from the secondary and tertiary alcohols or could be due to steric hindrance. To support this hypothesis the reaction was carried out under optimised reaction conditions with 50 mole% of MP-SO_3_H-8 catalyst. We observed the complete conversion of secondary alcohol (cyclohexanol) with exclusive selectivity to cyclohexene. The similar results have been observed in the pioneering work reported by Clark et. al.^22^. The benzylic alcohol demonstrated the good yield of alkyl methyl carbonate under similar reaction conditions (Table 3). The study shows that the material can be successfully employed to transform various primary, secondary and benzylic alcohols into corresponding carbonate esters. The methodology developed herein is simple and more efficient for the carboxymethylation of alcohols with DMC over MP-SO_3_H as compared to the first solid acid catalyst reported for this reaction Table S5.

**Table 3:** Substrate scope study.

Substrate (R-OH)	Alcohol conversion (mol %)	Selectivity to alkyl methyl carbonate (mol%)
Ethanol	95.3	97.0
Propanol	94.8	100.0
Iso-propanol	33.3	100.0
Butanol	100.0	96.6
Isobutanol	92.3	96.2
1-hexanol	100.0	98.6
Cyclohexanol	43.8	100
1-octanol	100.0	97.6
*Tert*-butanol	NC	–
Benzyl alcohol	59.6	80.6
Cyclohexanol	98.0	0.0^a,b^

Reaction conditions: Catalyst—MP-SO_3_H-8, catalyst concentration—5 mol% (w.r.t alcohol), mole ratio—1:12.5 (alcohol:DMC), reaction time—24 h, conversions were calculated with respect to the limiting reagent (alcohol), reflux condition, NC—no conversion.

^a^50 mol% catalyst, with 1:40 mol ratio.

^b^> 99% selectivity to cyclohexene.

The carboxymethylation of alcohol was also performed in large scale to test the efficiency of MP-SO_3_H-8 for the synthesis of alkyl methyl carbonates. The batch size used for large scale synthesis is 18 times bigger than the actual batch size used for the experiments reported in this work. The large scale synthesis of alkyl methyl carbonates using MP-SO_3_H-8 demonstrated the similar yield as that of small batch which ensures that the catalyst is efficient to retain its higher catalytic activity even in large scale synthesis. The results of the large scale synthesis are given in Table S6.

The catalysts screened for the DMC mediated carboxymethylation of alcohols was taken out and washed thoroughly with methanol. Then the washed material was dried in hot air oven for the time period of 2 h at 100 °C and subjected for the analysis of nitrogen sorption, FTIR, SEM, TEM analysis and acid–base titrations. The BET analysis of spent catalyst (MP-SO_3_H-8) showed the similar specific surface area and pore dimensions and retained the original characteristic hysteresis loop (Table S7 and Figure S9). The FTIR analysis of the spent material was performed and no additional peaks were observed in the IR spectra (Figure S10). Acid–base titration was performed for the spent catalyst and the material showed similar acidity as the fresh catalyst (Table S7). The transmission electron microscopy analysis of spent catalyst (MP-SO_3_H-8) demonstrated the porosity which is similar to fresh catalyst (Figure S11). In addition, the scanning electron microscopy analysis of spent material showed the similar morphology and particle sizes (Figure S12). The spent catalyst analysis by nitrogen sorption, FTIR, SEM, TEM analysis and acid–base titrations confirmed that the material is stable under reaction conditions and can be used in successive runs without losing its activity.

## Conclusions

The mesoporous polymers were synthesized solvothermally and functionalised with –SO_3_H groups by post synthetic modification. The sulfonation time was varied in order to have different amount of acidity in the functionalised mesoporous polymers. The physico-chemical properties and catalytic activity results are well correlated. Among all the catalysts screened, the MP-SO_3_H-8 was the best catalyst in order to achieve the high selectivity for the desired product with good conversion of alcohols under the optimized reaction conditions. The intrinsic mesoporosity, high surface area, optimised acidity and uniform surface coverage of active sites makes MP-SO_3_H-8 as the potential candidate for the DMC mediate carboxymethylation of alcohols. General applicability of the present protocol was extended to various alcohols including primary, secondary, tertiary and benzylic alcohols to their corresponding alkyl methyl carbonates. More than 95% conversion of primary alcohols to yield alkyl methyl carbonate esters selectively was observed. The moderate conversions were observed for secondary alcohols with less amount of catalytic material and no conversions were observed for *tert*-alcohols. The good yield of benzyl methyl carbonates was achieved under similar reaction conditions. The catalyst recyclability study and hot filtration experiments showed MP-SO_3_H-8 is an efficient, recyclable and stable material. To the best of our knowledge, MP-SO_3_H-8 is the first solid acid catalyst reported to demonstrate high yield of alkyl methyl carbonates under mild reaction conditions. The approach employed herein is the greenest among the reported acid mediated carboxymethylation of alcohols till date.

## Experimental

### Chemicals

The chemicals and materials used in the DMC mediated carboxymethylation of alcohols were obtained from the commercial suppliers and used without further purification. Dimethyl carbonate (LobaChemie, 99.0%), n-butanol (Merck, 99.0%), tert-butanol (Merck, 99.0%), Iso-butanol (Merck, 99.0%), ethanol (Changshu Hongsheng Fine Chemicals, 99.9%), n-propanol (Merck, 99.0%), cyclohexanol (SDFCL, 95%), H_2_SO_4_ (Merck, 95–98%), dichloromethane (DCM) (Merck, 99.5%), methanol (Merck, 99.0%), 1-hexanol (Avra chemicals, 98.0%), 1-octanol (Avra chemicals), benzyl alcohol (Merck, 99.0%), divinylbenzene (DVB) (TCI chemicals), azobisisobutyronitrile (AIBN) (Paras polymers and chemicals, India) and silver sulfate (Lobachemie, 99.0%).

### Materials

The NH_4_-beta zeolite (SAR-25) was procured from Nankai, China and Amberlyst-15 was purchased from Alfa Aesar. The NH_4_^+^ form of zeolites received were converted to protonic form by calcination at 550 °C for 5 h with the heating rate of 5 °C/min.

### Synthesis of functionalized mesoporous polymer (MP-SO_3_H)

The mesoporous polymer (MP-SO_3_H) was prepared by following the procedure reported in the literature^56^. The mixture containing 4 g DVB (divinylbenzene), 40 ml tetrahydrofuran, 4 g water, and 0.1 g AIBN (azobisisobutyronitrile) was taken in a round bottom flask. The flask was closed with glass stopper and stirred it at 30 °C for 3 h using stir bar. The homogeneous mixture obtained was hydrothermally treated at 100 °C for 48 h in a teflon lined autoclave with stainless steel jacket. After cooling down to room temperature, the polymer was crushed and solvent was allowed to evaporate slowly for 2 days. The final powdered material was outgassed at 100 °C for 3 h and labeled it as MP (mesoporous polymer).

The mesoporous polymer (MP) was used as the support for the anchoring of –SO_3_H groups. As a typical run, 1.5 g of MP was taken in a round bottom (RB) flask containing 30 ml DCM, 70 ml of conc. H_2_SO_4_ and 20 mg of Ag_2_SO_4_ catalyst. The mixture was stirred at 80 °C for the desired time period (4 h, 8 h, 12 h, and 24 h). The sulfonated mesoporous polymer (MP-SO_3_H) obtained was filtered and washed with excess of water and methanol. The washed material was dried at 100 °C for 8 h in a hot air oven and designated as MP-SO_3_H-*X* (X = 4 h, 8 h, 12 h, 24 h).

For comparison, the conventional materials such as Ambelyst-15, HBEA, and sulfated zirconia are screened for the carboxymethylation of alcohols with DMC.

### Characterization

The FTIR analysis of all the polymeric materials has been performed using alpha T-Bruker spectrophotometer in transmittance mode (450 cm^−1^ to 4,000 cm^−1^). The IR analysis by KBr pellet method was used to confirm the presence of Brønsted acidic sulfonic sites in the material. The surface area and kind of porosity of the samples were determined by nitrogen adsorption–desorption measurement using BELSORP-mini-II instrument at liquid nitrogen temperature (− 196 °C). The acidity of the polymer catalysts was determined by acid–base titration using standard 0.01 N NaOH solution. As a typical procedure, the 100 mg of catalyst (MP-SO_3_H-X) was allowed to ion-exchange with 10 ml of 2 N NaCl solution for 8 h. The liberated HCl was titrated against the known concentration of NaOH solution using phenolphthalein indicator. Similarly, the acidity of Amberlyst-15 was calculated. The composition of the polymeric catalyst was studied by elemental analysis (CHNS). The acidity values obtained by elemental analysis and titration are in good agreement with each other. Thermogravimetric analysis (TGA) has been carried out in air flow from 30 to 800 °C at the ramp rate of 10 °C/min to understand the thermal stability of the material. The morphology of polymer samples and elemental mapping has been performed using Hitachi SU 3500N SEM–EDX instrument. TEM and TEM-EDS analysis has been performed to get information about porosity and mode of distribution of the active sites over the material. The structure and functionality of the material is confirmed using ^13^C NMR analysis. The surface elemental composition of MP-SO_3_H has been characterized by XPS analysis. X-ray diffraction (XRD) patterns of the MP-SO_3_H samples were recorded on X-ray diffractometer (Bruker D-2 Phaser) with Cu k-alpha source having radiation wavelength of 0.1546 nm through a 2θ range from 5° to 90°.

### Catalytic reaction

Catalytic experiments were performed in a 25 ml single neck round bottom flask fitted with reflux condenser and equipped with magnetic stirrer. The reflux condenser temperature was maintained at 8 °C using water circulating cryostat to prevent the evaporation of reactants. The required amount of alcohol (6.0 mmol) and dimethyl carbonate (75.0 mmol) were taken in round bottom flask. The alcohol and DMC are mixed properly by stirring it at room temperature for 10 min followed by the addition of preactivated polymer catalyst (5 mol% w.r.t alcohol). Then the reaction mixture was heated to 90 °C and maintained at the same temperature for the time period of 24 h with constant stirring. After completion of each catalytic reaction, the oil bath was allowed to cool down to room temperature. The samples were withdrawn and quantification of the products have been performed using gas chromatograph (Agilent 7820A) connected with HP-5 capillary column (0.25 mm I.D. 30 m) equipped with flame ionization detector. The conversion and selectivities were calculated by using formula.

$$\% {\text{ Conversion of Alcohol }} = \frac{{{\text{Initial moles of alcohol }}{-}{\text{ Final moles of alcohol}}}}{{\text{Initial moles of alcohol}}} \times 100$$

$$\% {\text{ Selectivity }} = \frac{{\text{Moles of desired product}}}{{\text{Moles of alcohol converted}}} \times { 1}00$$

## Supplementary information

Supplementary Information
