# Supplementary material for: Solid acid catalyzed carboxymethylation of bio-derived alcohols: an efficient process for the synthesis of alkyl methyl carbonates
Source: Sci Rep. 2020 Aug 4;10:13103. doi: 10.1038/s41598-020-69989-7 (PMC7403395; doi:10.1038/s41598-020-69989-7)
Supplement: Supplementary file 1 — Supplementary Information [file 41598_2020_69989_MOESM1_ESM.docx]

**Supporting Information**

**Solid acid catalyzed carboxymethylation of bio-derived alcohols: An efficient process for the synthesis of alkyl methyl carbonates**

**Kempanna S. Kanakikodi^1,2^, Sathyapal R. Churipard^1,2^, A. B. Halgeri^1^ and Sanjeev P. Maradur^1^***

*^1^ Materials Science & Catalysis Division, Poornaprajna Institute of Scientific Research (PPISR), Bangalore, Bidalur Post-562110, India.*

*^2^ Graduate studies, Manipal Academy of Higher Education, Manipal -576104, India.*

*Email:[**sanjeevpm@poornaprajna.org**](mailto:sanjeevpm@poornaprajna.org)

**Figures**

**Figure S1: Scanning Electron Microscopy analysis of MP and MP-SO_3_H**

**Figure S2: Adsorption-desorption isotherms of MP-SO_3_H-x**

**Figure S3: Contact angle images of a) MP and b) MP-SO_3_H-8**

**Figure S4: FTIR spectra of MP and MP-SO_3_H materials**

**Figure S5: TGA plots of MP-SO_3_H materials**

**Figure S6: TEM images of MP and MP-SO_3_H materials**

**Figure S7: The XRD pattern of the MP-SO_3_H**

**Figure S8: GC-MS analysis of carboxymethylation reaction mixture**

**Figure S9: Adsorption-desorption isotherm of spent MP-SO_3_H-8**

**Figure S10: FTIR spectra of spent MP-SO_3_H-8**

**Figure S11: Transmission electron micrograph of spent MP-SO_3_H-8**

**Figure S12: Scanning electron microscopy image of spent MP-SO_3_H-8**

**Tables**

**Table S1:** Surface composition of the polymer catalyst MP-SO_3_H-8

**Table S2:** Acidity of all the materials used in DMC mediated carboxymethylation

**Table S3:** Physico-chemical properties of HBEA, Amberlyst-15 and SO_4_^-2^/ZrO_2_

**Table S4:** Results of standard deviation

**Table S5:** Comparison of catalytic activity of MP-SO_3_H-8 with NZSM-5

**Table S6:** Results of large scale carboxymethylation of butanol

**Table S7:** Physico-chemical properties of spent catalyst

**Figure S1: Scanning Electron Microscopy analysis of MP and MP-SO_3_H**

**
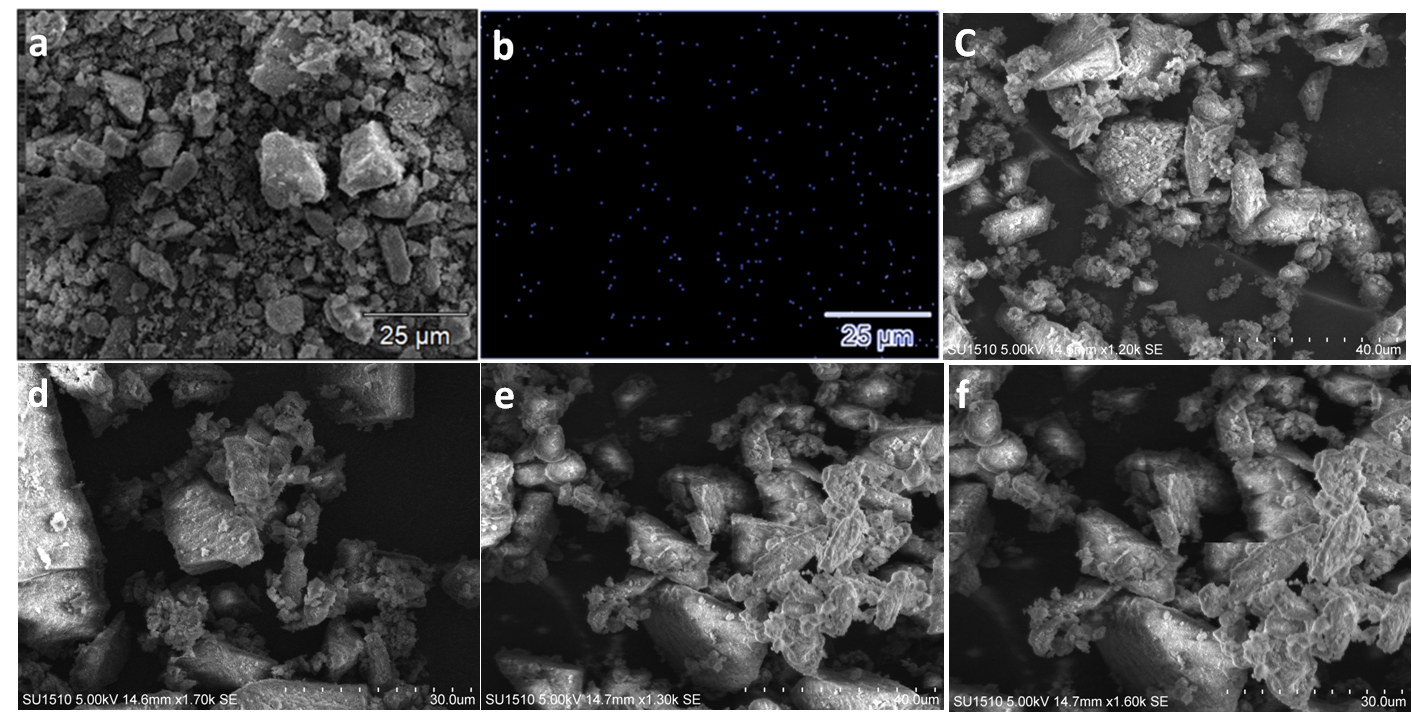
**

**SEM images of a) MP-8, b) SEM EDX of MP-8, c) MP, d) MP-4, e) MP-12, f) MP-24**

**
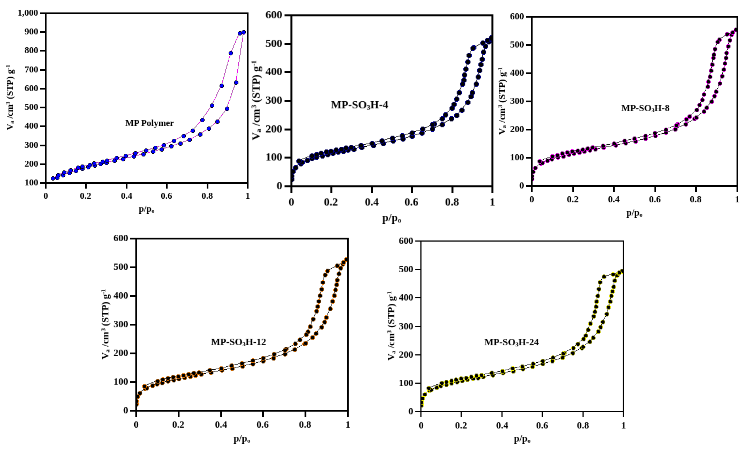
Figure S2: Adsorption-desorption isotherms of MP-SO_3_H-x**


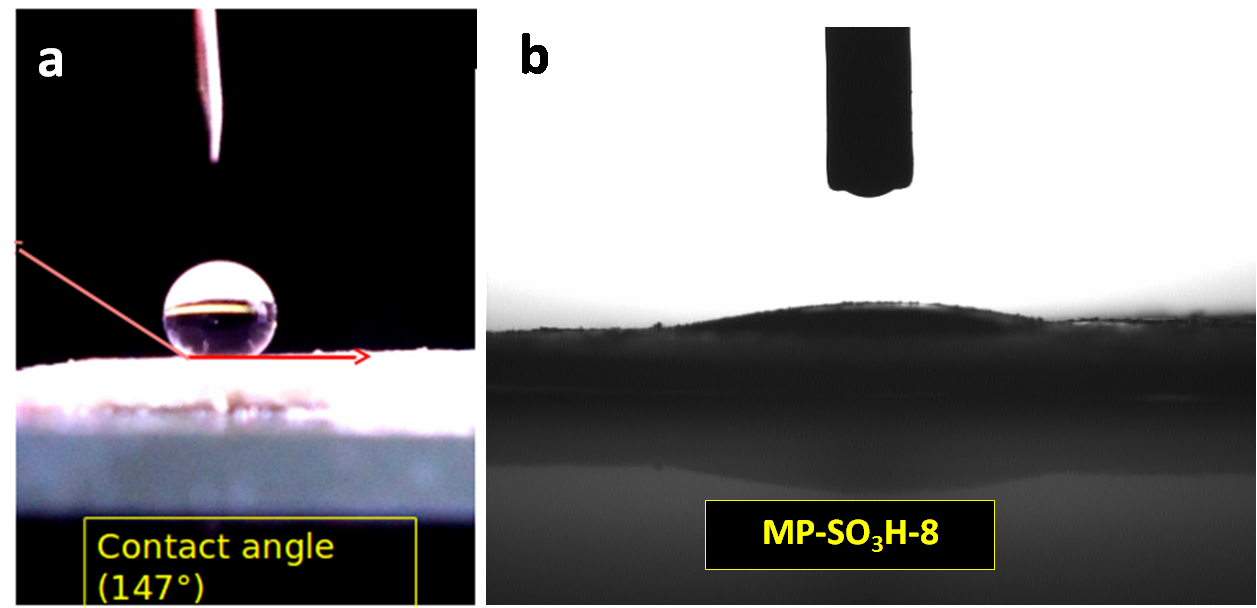
**Figure S3: Contact angle images of a) MP and b) MP-SO_3_H-8**

**a) Contact angle of water droplet on MP surface, b) Contact angle of water droplet on MP-SO_3_H-8 surface**

- Image a) indicates that MP is highly hydrophobic material but by the sulfonation of MP lead to the decrease in the surface area, pore dimensions and more importantly which imparts the hydrophilicity in the material. The image (b) indicates the hydrophilic nature of the material which does not form any angle with the catalyst surface.

**Figure S4: FTIR spectra of MP and MP-SO_3_H materials**


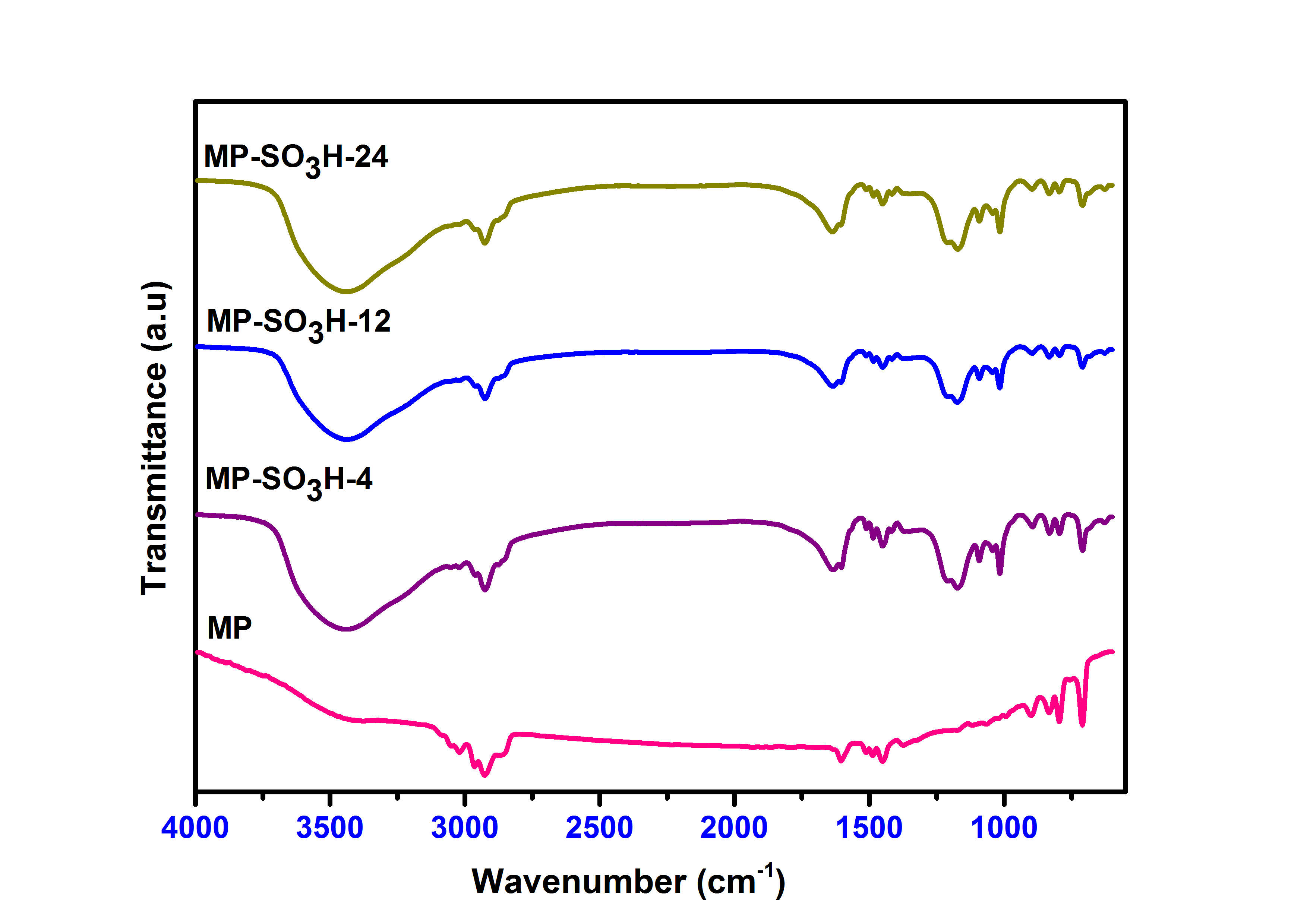


**Figure S5: TGA plots of MP-SO_3_H materials**


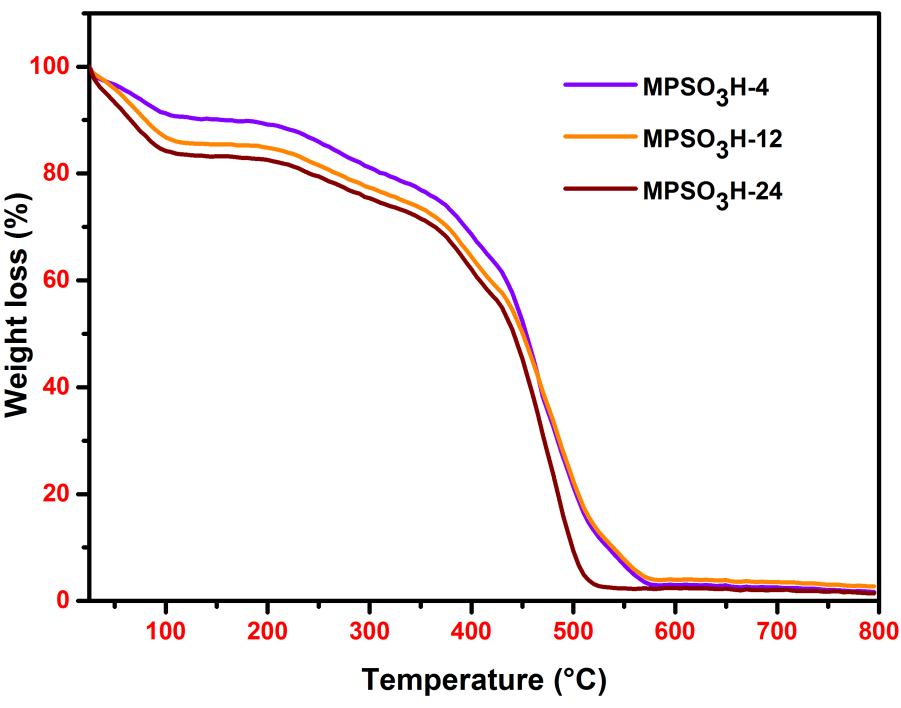


**Figure S6: TEM images of MP and MP-SO_3_H materials**


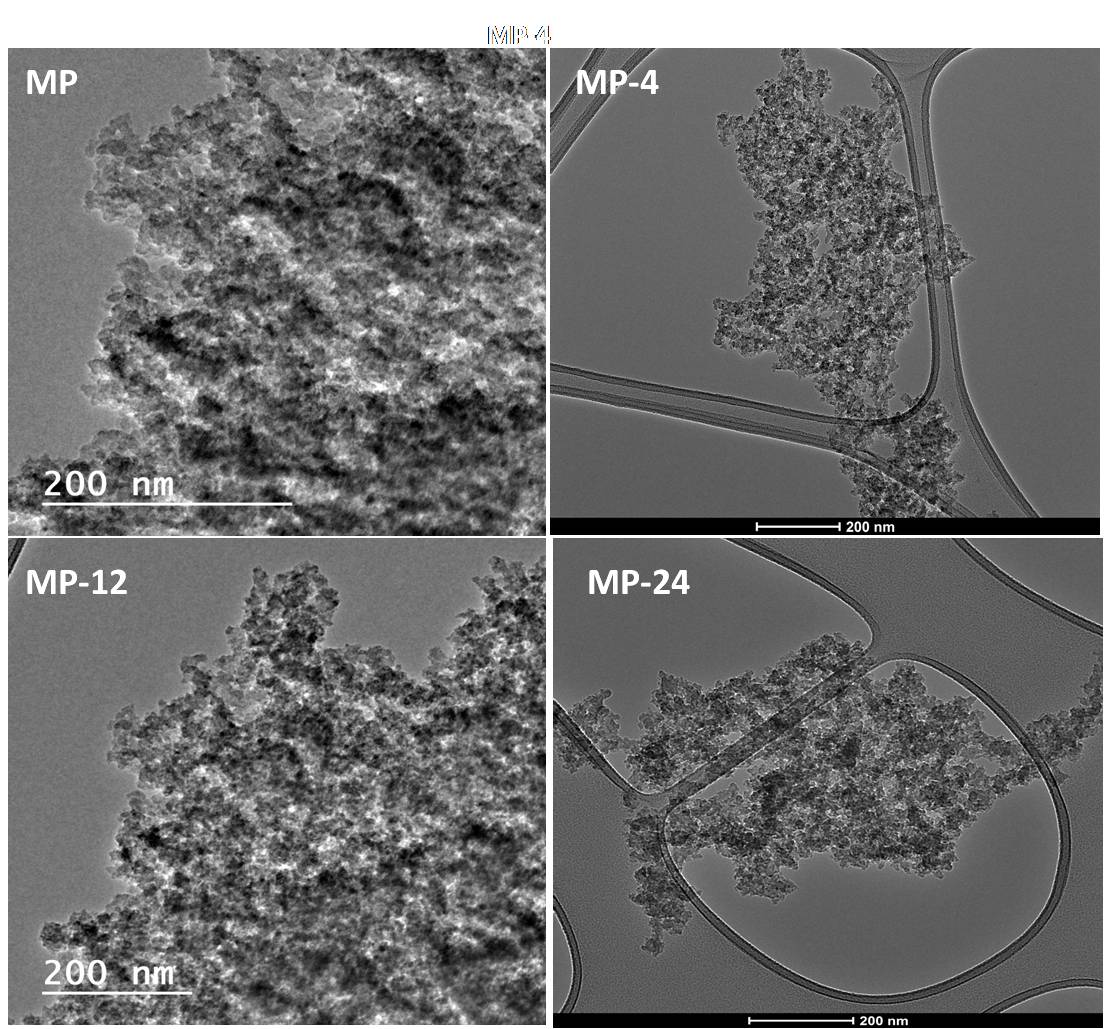


**Figure S7: The XRD pattern of the MP-SO_3_H**

**
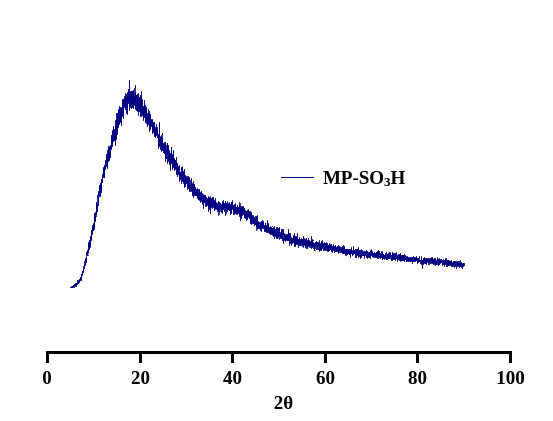
**

**Figure S8: GC-MS analysis of carboxymethylation reaction mixture**

**
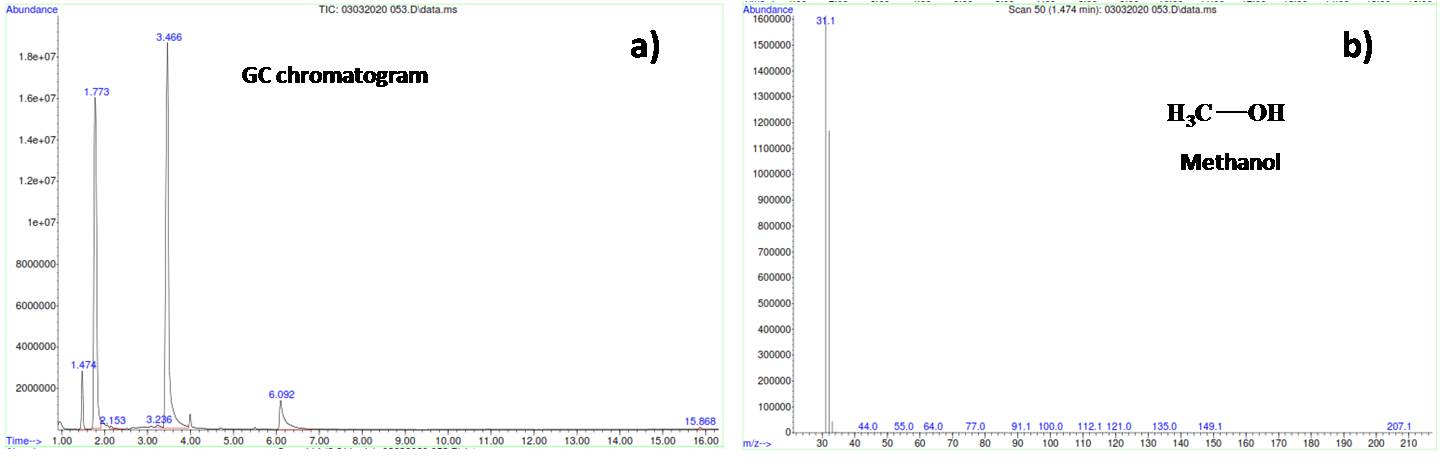
**

**
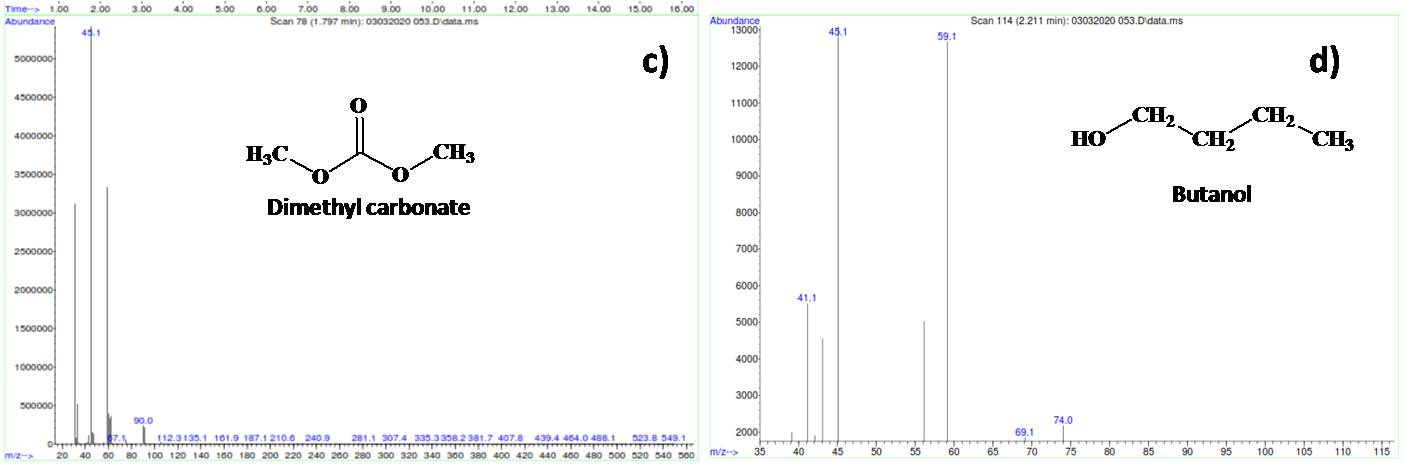
**

**
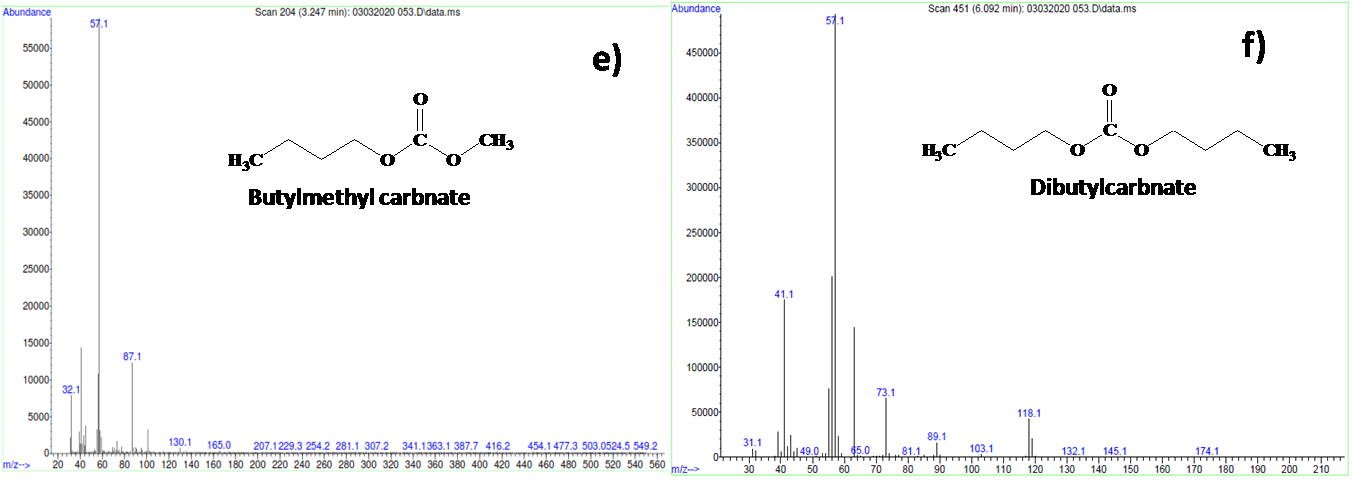
**

1. **Chromatogram of reaction mixture**
2. **Mass fragmentation pattern of methanol**
3. **Mass fragmentation pattern of dimethyl carbonate**
4. **Mass fragmentation pattern of butanol**
5. **Mass fragmentation pattern of butyl methyl carbonate**
6. **Mass fragmentation pattern of dibutyl carbonate**

**Figure S9: Adsorption-desorption isotherm of spent MP-SO_3_H-8**

**
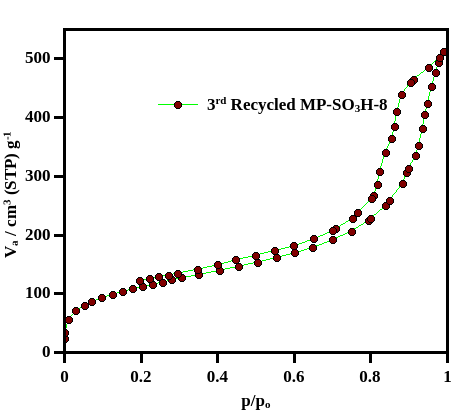
**

**Figure S10: FTIR spectra of spent MP-SO_3_H-8**


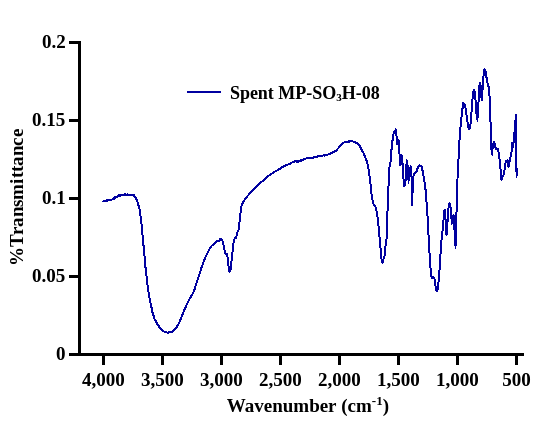


**Figure S11: Transmission electron micrograph of spent MP-SO_3_H-8**





**Figure S12: Scanning electron microscopy image of spent MP-SO_3_H-8**


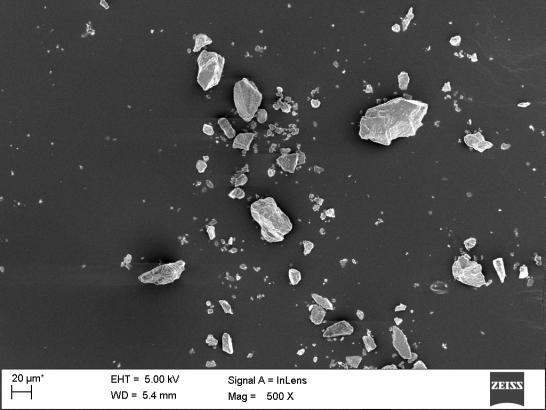


**Table S1: Surface composition of the polymer catalyst MP-SO_3_H-8**

| **Name** | **Peak (BE)** | **Atomic %** |
| --- | --- | --- |
| O1s | 533.1 | 34.13 |
| C1s | 286.4 | 53.82 |
| S2p | 169.5 | 5.39 |
| C1s | 284.8 | 6.66 |

**Table S2: Acidity of all the materials used in DMC mediated carboxymethylation**

| Sl. no. | Catalyst | Acidity mmol H^+^/g |
| --- | --- | --- |
| 01 | MP-SO_3_H-04 | 1.7^a^ |
| 02 | MP-SO_3_H-08 | 1.9^a^ |
| 03 | MP-SO_3_H-12 | 2.0^a^ |
| 04 | MP-SO_3_H-24 | 2.3^a^ |
| 05 | Amberlyst-15 | 4.7^a^ |
| 06 | SO_4_^-2^/ZrO_2_ (2N) | 1.4^b^ |
| 07 | HBEA (25) | 1.5^b^ |

^a^= Acidity measured by acid base titrations

^b^= From literature

**Table S3: Physico-chemical properties of HBEA, Amberlyst-15 and SO_4_^-2^/ZrO_2_**

| **Sample** | **S_BET_(m^2^ g^-1^)** | **V_t_ (ccg^-1^)** | **Pore size^a^ (nm)** | **Acidity (mmol/g)** |
| --- | --- | --- | --- | --- |
| HBEA | 465 | - | - | 1.51^b^ |
| Amberlyst-15 | 39 | - | 23.3 | 4.7 ^c^ |
| SO_4_^-2^/ZrO_2_ | 57 | - | 2.3 | 1.4 ^b^ |

*The physicochemical properties of HBEA, Amberlyst-15, and SO_4_^-2^/ZrO_2_ are taken from the literature

^a^ = Pore size distribution by BJH method

^b^ = From NH_3_ TPD Analysis

^c^ = Measured by acid-base titration

**Table S4: Results of standard deviation**

| **Repeatability** | **Catalyst** | **Butanol**  **Conversion (%)** | **% Selectivity**  **(BMC)** |
| --- | --- | --- | --- |
| 1 | MP-SO_3_H-8 | 91.8 | 94.7 |
| 2 | MP-SO_3_H-8 | 92.2 | 93.6 |
| 3 | MP-SO_3_H-8 | 91.5 | 94.3 |

**Reaction Conditions:** Catalyst concentration- 5mol% (w.r.t Butanol), mole ratio- 1:10 (butanol:DMC), reflux condition, reaction time- 24h, conversions were calculated with respect to the limiting reagent (Butanol), BMC (butyl methyl carbonate).

* The standard deviation in the repeated experiments were less than 1%

**Table S5: Comparison of catalytic activity of MP-SO_3_H-8 with NZSM-5**

| **Catalyst** | **Temp. (°C)** | **Catalyst**  **(wt%)** | **BuOH:DMC** | **Time (h)** | **Mode of reaction** | **Butanol**  **Conv. (%)** | **% Selec.**  **(BMC)** |
| --- | --- | --- | --- | --- | --- | --- | --- |
| MP-SO_3_H-8 | 90 | 35 | 1:12.5 | 24 | RB with reflux | >99.9 | 95.5 |
| NZSM-5 | 110 | 110 | 1:23 | 24 | Sealed tube | - | 91% yield |

**Table S6: Results of large scale carboxymethylation of butanol**

| **Catalyst** | **Butanol**  **Conversion (%)** | **% Selectivity**  **(BMC)** |
| --- | --- | --- |
| MP-SO_3_H-8 | 95.4 | 97.1 |

**Reaction Conditions:** Catalyst concentration - 5mol% (w.r.t Butanol), Butanol - 108mmoles DMC-1350mmoles, reflux condition, reaction time- 24h, conversions were calculated with respect to the limiting reagent (Butanol), BMC (butyl methyl carbonate).

**Table S7: Physico-chemical properties of spent catalyst**

| **Sample** | **S_BET_(m^2^ g^-1^)** | **V_t_ ^a^(ccg^-1^)** | **d_ave_ (nm)** | **S content ^b^ (mmol/g)** | **Acidity ^c^**  **(mmol/g)** |
| --- | --- | --- | --- | --- | --- |
| MP-SO_3_H-8^d^ | 409 | 0.72 | 7.7 | - | 1.87 |

^a^ = from BJH method,

^b^ = Measured by elemental analysis,

^c^ = Measured by acid-base titration,

d_ave_ = Average pore diameter from BET

d = 3^rd^ Recycled catalyst
